# Optical Fibre NO_2_ Sensor Based on Lutetium Bisphthalocyanine in a Mesoporous Silica Matrix

**DOI:** 10.3390/s18030740

**Published:** 2018-03-01

**Authors:** Marc Debliquy, Driss Lahem, Antonio Bueno-Martinez, Christophe Caucheteur, Marcel Bouvet, Isaline Recloux, Jean-Pierre Raskin, Marie-Georges Olivier

**Affiliations:** 1 Service de Science des Matériaux, Faculté Polytechnique, Université de Mons, 7000 Mons, Belgium; isaline.recloux@umons.ac.be (I.R.); marjorie.olivier@umons.ac.be (M.-G.O.); 2 Materia Nova, Materials R&D Centre, Parc Initialis, Avenue Nicolas Copernic 1, 7000 Mons, Belgium; driss.lahem@materianova.be; 3 Service d’Electromagnétisme et de Télécommunications, Faculté Polytechnique, Université de Mons, 7000 Mons, Belgium; antonio.buenomartinez@umons.ac.be (A.B.-M.); Christophe.caucheteur@umons.ac.be (C.C.); 4 Institut de Chimie Moléculaire de l’Université de Bourgogne, UMR CNRS 6302 Univ. Bourgogne Franche-Comté, 21078 Dijon, France; marcel.bouvet@u-bourgogne.fr; 5 Institut ICTEAM, Université catholique de Louvain-la-Neuve, 1348 Louvain-la-Neuve, Belgium; jean-pierre.raskin@uclouvain.be

**Keywords:** optical fibre sensors, sol-gel, nitrogen dioxide, lutetium bisphthalocyanine

## Abstract

In this article, we describe a NO_2_ sensor consisting of a coating based on lutetium bisphthalocyanine (LuPc_2_) in mesoporous silica. The sensor exploits the absorption spectrum change of this material which strongly and reversibly decreases in contact with NO_2_. NO_2_ is measured by following the amplitude change in the reflected spectrum of the coating deposited on the tip of a silica fibre. As diffusion of NO_2_ in LuPc_2_ is slow, the response time could be slow. To reduce it, the active molecules are dispersed in a mesoporous silica matrix deposited by a sol-gel process (Evaporation Induced Self Assembly) avoiding the formation of large crystals. Doing so, the response is fairly fast. As the recovery is slow at room temperature, the recovery time is reduced by exposure to UV light at 365 nm. This UV light is directly introduced in the fibre yielding a practical sensor sensitive to NO_2_ in the ppm range suitable for pollution monitoring.

## 1. Introduction

The combustion of fossil fuels leads to the massive release of CO_2_, SO_2_, CO and other pollutants such as nitrogen oxides (NOx) which are toxic, cause acid rain and production of ozone in low atmosphere [1]. Nowadays, the emission of internal combustion engines, especially Diesel engines, is the main source of NOx, especially NO_2_, which is one of the main toxic gases that may cause respiratory and coronary diseases [2]. 

The pollution problem is more acute in confined places like tunnels or car parks. That is why an efficient monitoring system should be developed for these specific cases. Results of studies in which people have been exposed to NO_2_, have demonstrated that this gas can negatively affect healthy people as well as sensitive people. For healthy people, effects have been observed for peak levels higher than 4000 μg/m^3^ (2 ppm); no effects have been observed for peak levels below 2000 μg/m^3^ (1 ppm) [3]. Based on these findings, PIARC proposed an in-tunnel air quality level of 1 ppm NO_2_ [3] as an average value. Some countries have introduced values for different time frames. For very short time considerations, France adopted the WHO threshold value and proposes 0.4 ppm (as an average over 15 min). However, the WHO limit aims at improving the air-quality in general and is not intended to be applied as peak exposure. Based on recent studies, Sweden is currently in the process of abandoning the WHO threshold as in-tunnel air-quality limit [4]. The experimental study by Langrish et al. [5] showed that a 4 hr exposure to 8000 μg/m^3^ (4 ppm) did not give any significant vascular effect by the participants. Belgium applies 1000 μg/m^3^ (as an average over 20 min) and Norway 1.5 ppm (as an average over 15 min) [6] as the limiting concentration inside a tunnel. Many countries do not apply a limit to NO_2_ for tunnel users but the short-time working exposure limit (e.g. 3.0 ppm in Switzerland, 5 ppm as US NIOSH standard) implicitly applies. The typical requirements for this application are: concentration range (0–5 ppm), response time <15 min. 

Optical systems based on optical fibres can be very useful in those specific applications. Various techniques exist which are all based on the deposition of a sensitive layer reacting with the gases, changing its optical properties (complex refractive index). Let us cite, for example, the deposition of a sol-gel coating on the core of a fibre [7], on a fibre with a reduced diameter [8], on fibre Bragg gratings [9,10] or on the tip of a fibre [11,12]. One of the first configurations was based on modified porous silica fibre presenting changes of the transmission spectra under NO_2_ exposure [13]. In other works, the sensor element consisted in the replacement of a portion of the cladding region of a multimode plastic clad silica fibre by metallophthalocyanines such as CuPc, PbPc and SmPc [14]. On the other hand, some other works describe extrinsic configurations. In that case, the fibre is only used to transmit/receive the optical signal after the reflection in the sensitive material immobilized in a separate membrane (deposited in a sol-gel film [15] or a disc [16] or after transmission passing through a sensing plate [17]).

In this paper, we present a NO_2_ optical fibre sensor based on a coated fibre tip, using LuPc_2_ as a sensitive molecule encapsulated in a mesoporous silica matrix. 

The response of the sensor is based on the decrease of absorption at 660 nm following NO_2_ adsorption. The adsorption is reversible but very slow at room temperature. Indeed, when pure LuPc_2_ is used, as the diffusion of the gases is very slow, the response times can be terribly long [18]. A solution can be to use very thin films like in [19]. Here, we propose to encapsulate LuPc_2_ in a mesoporous structure that consists in pores of a few nm filled with LuPc_2_. The grains are limited in size and the intracrystalline diffusion then fast results in a reduced response time. The mesoporous matrix consists of a silica matrix deposited by a specific sol-gel method—EISA (Evaporation Induced Self Assembling)—starting from TEOS and MTES as precursors and Pluronic^R^ P123 as templating agent. After removal of the template by calcination, this matrix with high porosity and specific surface area is impregnated with LuPc_2_ by dipping in a chloroform solution. This process is simple and well adapted for optical fibres. 

Moreover, to shorten the recovery time, the films were exposed to ultraviolet (UV) light at 365 nm during the measurements. The role of UV light is to accelerate the desorption of NO_2_. In order to get a practical system and to avoid the use of an external source for UV, both UV and red lights were injected in the same fibre with a configuration similar to the one described in our previous work [19].

## 2. Materials and Methods

### 2.1. Sensitive Material

Owing to their exceptional optical, electronic and electrochemical properties [20,21,22], metallophthalocyanines have attracted considerable interest. In particular, these organic molecules, because of the presence of delocalized π electrons, are sensitive to oxidizing or reducing gases at ppm concentrations. Indeed, the adsorption of gases presenting a redox character can involve an electron transfer during the interaction with these molecules leading to a change of the charge carrier number and as a consequence a change of the conductivity. This electron transfer can also lead to a change of the optical spectrum. As the interaction with the gases is reversible, phthalocyanines were studied as sensitive elements for conductive and optical gas sensors [23,24,25,26,27]. Another important characteristic for its use in gas detection is the remarkable chemical and thermal stability of the phthalocyanine derivatives.

**Figure 1:**
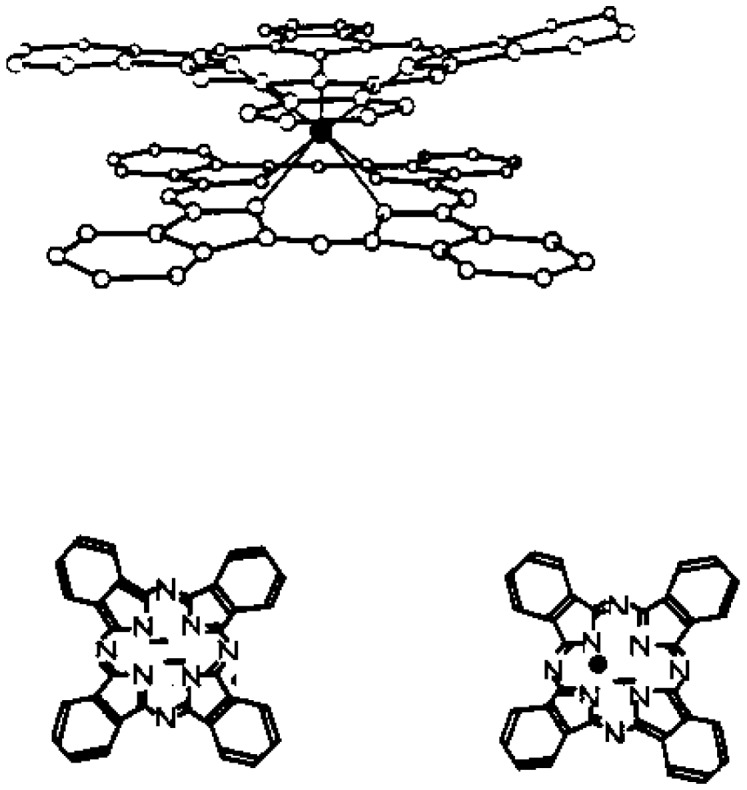
Molecular structure of lutetium bisphthalocyanine (LuPc_2_).

Lanthanide bisphthalocyanine (LnPc_2_) complexes with a “double-decker” structure [28], [LnPc_2_]-, are a typical class of compounds with π-π* transitions and they exist in different forms associated with different colours. The study of lanthanide diphthalocyanines is very attractive due to their electrochromic [29] properties and semiconducting [30,31] behaviours. Among these, the bisphtalocyanine of lutetium—LuPc_2_—is the model compound (Figure 1).

The low ionization energy together with the high polarization energy facilitates the transfer of charge between LuPc_2_ and acceptor molecules. The transfer of electrons will have an impact in the absorption spectra of LuPc_2_ in the UV, visible and near infrared range or on the conductivity. These molecules were then studied for gas detection [18,32,33,34].

The interaction of NO_2_ with LuPc_2_ molecules is described in expression (1). This electron transfer is reversible.

(1) $${\mathrm{LuPc}}_{2}+{\mathrm{NO}}_{2}↔{\mathrm{LuPc}}_{2}^{+}+{\mathrm{NO}}_{2}^{-}$$

The oxidation of LuPc_2_ induces a decrease of the Q-band absorption at 660 nm turning it from green to red.

Lutetium bisphthalocyanine (LuPc_2_) was synthetized from the o-dicyanobenzene by heating with lutetium triacetate Lu(OAc)_3_, at 300 °C, without any solvent according to a previously published procedure [35].

### 2.2. Mesoporous Matrix

The synthesis of ordered mesoporous films and their use for different applications such as catalysis, drug delivery, sensors, low-k dielectrics and other electro-optical technologies have been reported in many papers [36,37,38,39]. These materials are obtained by using surfactant molecules, which act as templates during the synthesis of the sol-gel film, through the Evaporation Induced Self-Assembly (EISA) process. 

In brief, [40] the principle is to get a solution of a surfactant molecule with a long hydrophobic tail and a hydrophilic head mixed with precursors of the desired oxide material. During the hydrolysis of the precursors, particles of the sol will interact with the surfactant. During the deposition process by dip-coating, the solvent evaporates and the critical micellar concentration is reached provoking the aggregation of the surfactant in micelles. The distribution of the dimensions of the micelles is very narrow and depends on the size of the hydrophobic tails. Working at high humidity in the atmosphere and so reducing the drying speed allows the micelles to get organized in a periodic lattice. The obtained structure depends on the concentration of the surfactant. The transformation of the mesostructured film into mesoporous film is then achieved by eliminating the templating agent from the sol-gel matrix. It is usually carried out by calcination (thermal treatment with a slow heating rate up to a plateau temperature around 400 °C) or solvent extraction [41]. The attractiveness of these films is mainly due to their ordered nanometric porosity (2–50 nm) and to their high specific surface area. Furthermore, the flexibility of the synthesis process allows getting film properties adapted for each application. Films with controlled pore size, orientation and connectivity can be achieved.

Glass fibres are made of silica (with an eventual dopant) and, logically, we chose the silica sol-gel coatings which present a good adhesion to silica. The mesoporous silica sol-gel coatings were proved to be resistant and are used as barrier layers to protect metals against corrosion [42].

In this work, silica mesostructured films have been synthesized through the EISA process using Pluronic P123 as a templating agent. The pore size distribution of films has been measured for each condition of removal by means of adsorption porosimetry using a quartz crystal microbalance as explained in [42].

The precursor solution was prepared by mixing absolute ethanol (Merck), Pluronic P123 (PEO)_20_-(PPO)_70_-(PEO)_20_ (Sigma-Aldrich, St. Louis, MO, USA) as templating agent, a mixture of tetraethylorthosilicate TEOS (Merck, Kenilworth, NJ, USA) and methyltriethoxysilane MTES (Merck) (75%/25%) and concentrated hydrochloric acid (Baker, Eupen Belgium, 36 wt %). In molar ratio, the composition was 1 TEOS/MTES, 0.005 P123, 6 H_2_O, 0.001 HCl and 9 Ethanol. The use of a mixture TEOS/MTES avoids the appearance of cracks during drying of deposited films. The quantity of water was chosen in order to promote hydrolysis of the alkoxide species and the pH value was set at 4 in order to slow down condensation reactions. The sol was left to react under stirring for 30 min at room temperature. 

Films were deposited in the same conditions on the tip of fibres and on flat glass substrates for easier characterization. Films were also deposited on quartz crystal microbalance substrates for porosity measurements according to [42]. 

In order to promote the adhesion of the mesoporous gel on the tip of the fibre cut at right angle, the fibres were cleaned with ethanol and then dipped for 2 h in HNO_3_ 1 M. This treatment favours the formation of silanol (Si-OH) groups on the surface that act as anchor points for the formation of the sol-gel.

Mesostructured silica films were deposited by dip-coating in a climatic chamber with controlled humidity and temperature (70% RH/25 °C), with a withdrawal rate of 60 mm/min. After dip-coating, the films were maintained in the deposition chamber in the same conditions of temperature and high humidity for 24 h. Working in a humid environment (>50% RH) promotes the periodic organization of micelles under mesophases inside the film [43]. 

The last step of the synthesis consists in removing the templating agent by calcination at 400 °C. The calcination treatment was performed in a furnace under air during 4 h with a temperature slope of 1 °C/min. We observed however that the annealing procedure is not a critical step impacting the results. 

The thickness of the films on the flat substrates was measured by a profilometer (Nanojura, Besançon, France) and found to be 320 ± 15 nm after calcination.

### 2.3. Impregnation with LuPc_2_

The mesoporous films were dipped in a saturated solution of LuPc_2_ in chloroform.

The amount of LuPc_2_ molecules in the matrix was evaluated using a quartz crystal microbalance and the optical absorption of the films like explained in the next paragraphs.

### 2.4. Measurement Setup for Gas Sensing Characterization

**Figure 2:**
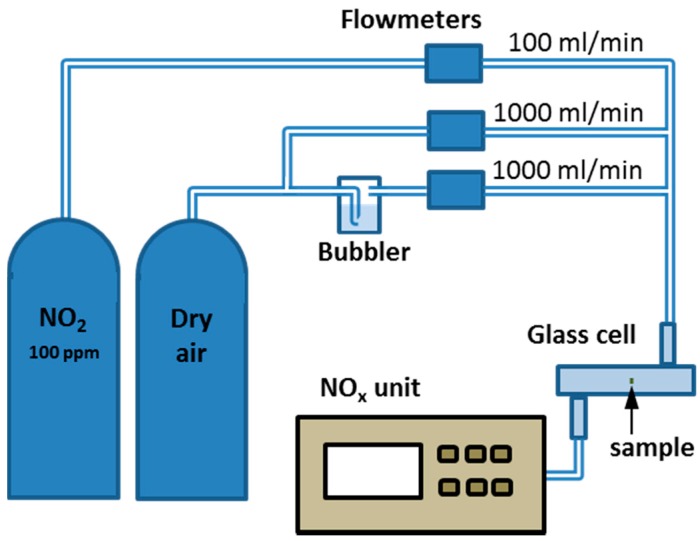
Gas test bench.

For the optical characterization of the sensitive layers towards gas mixtures, a gas test bench was used (Figure 2). The test bench consists of a set of mass flowmeters allowing to impose the humidity (by bubbling through a bubbler kept at 20 °C) and the NO_2_ concentration (by dilution from a bottle of 100 ppm NO_2_ in air). The NO_2_ concentration in the cell was controlled by means of a NOx chemiluminescence analyser (Thermo Environmental Instrument, Breda, The Netherlands).

**Figure 3:**
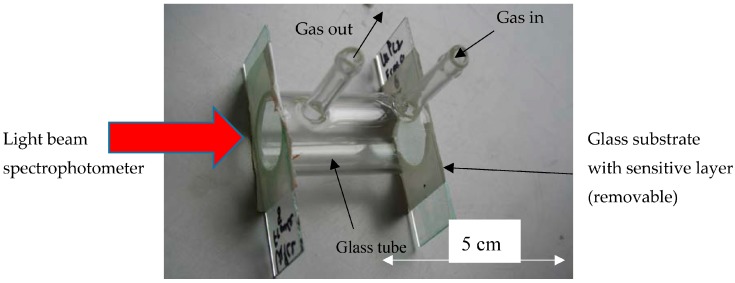
Cell used for the optical characterization with the gases on flat substrates.

For the optical characterization on flat substrates, a cell like shown in Figure 3 was used. The gas mixture was flown through the small tubes on Figure 3. One of the windows is removable and consists in the glass substrate covered with the sensitive layer glued on the cell (with a double-sided tape). This cell is placed in a UV-Vis spectrometer (Perkin Elmer lambda19 used in transmittance) and flushed by the gas thanks to the same gas system as presented above. 

The experimental setup for NO_2_ measurement with optical fibres is similar to that used in reference [15]. It makes use of glass cell (diameter = 1cm, length = 15 cm) continuously flown by a 1 L/min gas mixture with controlled humidity. 

The optical fibre sensor consists of a multimode fibre (400 µm core) cleaved at a right angle and coated with the gasochromic layer. 

**Figure 4:**
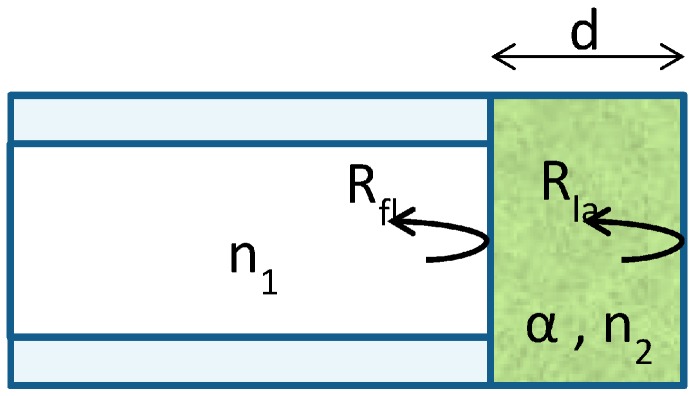
Scheme of the sensor.

Figure 4 presents a simple model for the fibre-based sensor.

Light is injected and the signal is the variation of the reflectance of the sensor or reflected power. Indeed, light is absorbed during the travel in the sensitive layer. In air without NO_2_, the absorption is maximal and the reflected power minimal. In contact with NO_2_, the absorption is reduced and the reflected power increases. A more detailed model is explained in Section 3.4. 

**Figure 5:**
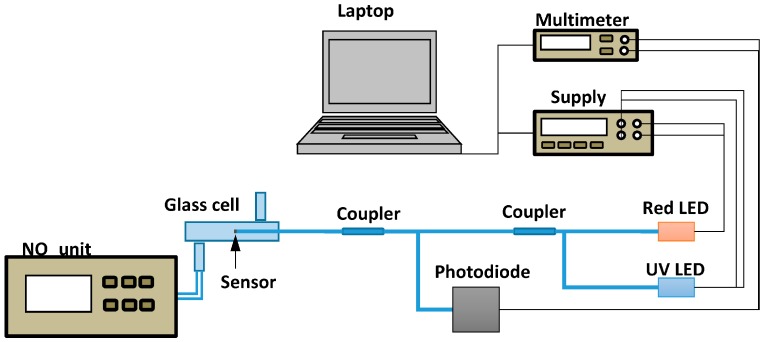
Set up for the measurements with optical fibre sensors.

For the optical measurements, two light-emitting diodes (LEDs) were used: a red LED emitting at 660 nm and a UV LED emitting at 365 nm (Figure 5). UV radiation is used to accelerate the recovery as was shown in reference [19]. 660 nm was chosen as it is close to the absorption maximum of LuPc_2_ as will be shown below. A 200 µm core diameter multimode optical fibre was connected to the UV LED (0.5 mW optical output power). On the other hand, a 400 µm diameter core multimode optical fibre was connected to the red LED (14.5 mW). Both LEDs were connected to an optical coupler with a coupling ratio 50:50 in order to combine the two wavelengths in the same fibre. The output of the coupler was connected to another coupler with identical characteristics. The sensitive fibre, a 400 µm diameter core multimode fibre covered with the sensitive coating at the fibre end, is connected to the output of the second coupler. The light reflected by the fibre end to the optical coupler is finally detected by a photodiode. Because UV light disturbs the electrical measurement, adding a superimposed voltage level, the source needs to be synchronized with the multimeter. When a measurement is to be done, the power supply of the UV LED is disabled and the voltage measured by the multimeter is registered. After that, the power supply of the UV LED is re-established. The power density at the end of the fibre is about 290 W/m^2^.

**Figure 6:**
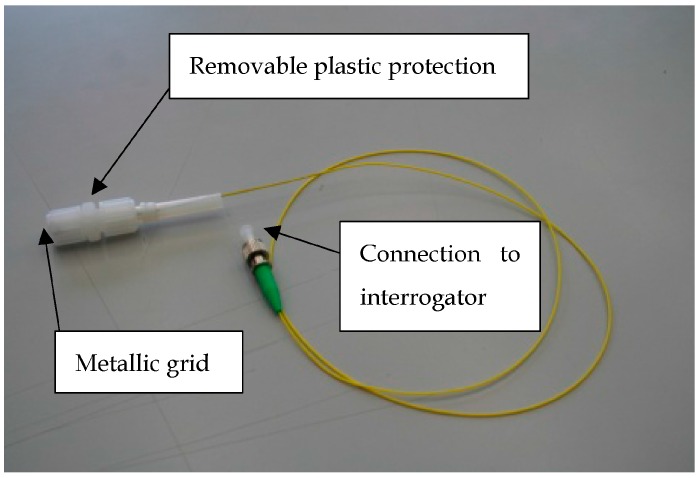
Picture of the sensor.

For the tests, the fibre tip is protected in a sheath fitted with a metallic grid (Figure 6). 

## 3. Results and Discussion

### 3.1. Characterization of the Mesoporous Matrix

**Figure 7:**
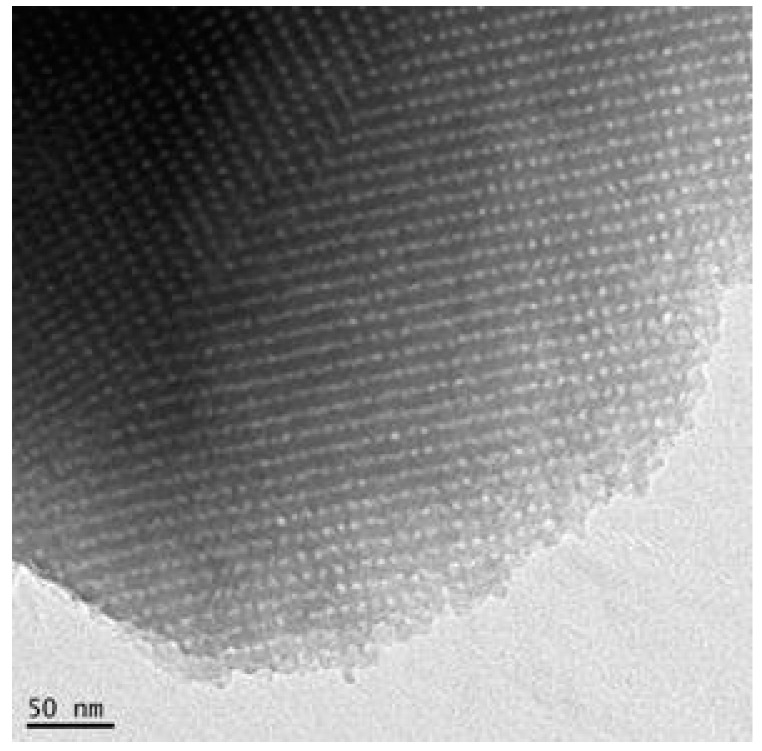
TEM image of a mesoporous silica film after calcination.

The films deposited on flat glass substrates were observed by TEM (Figure 7).

Regularly spaced pores (mean size 4 nm) can clearly be seen from the images.

**Figure 8:**
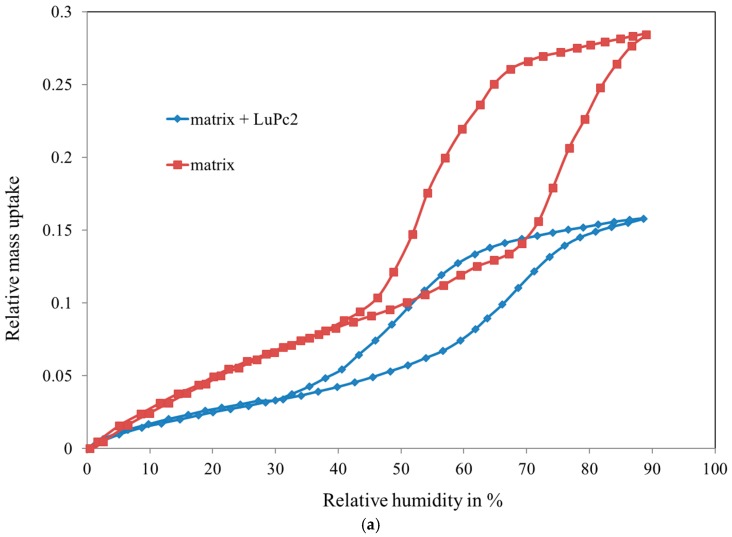

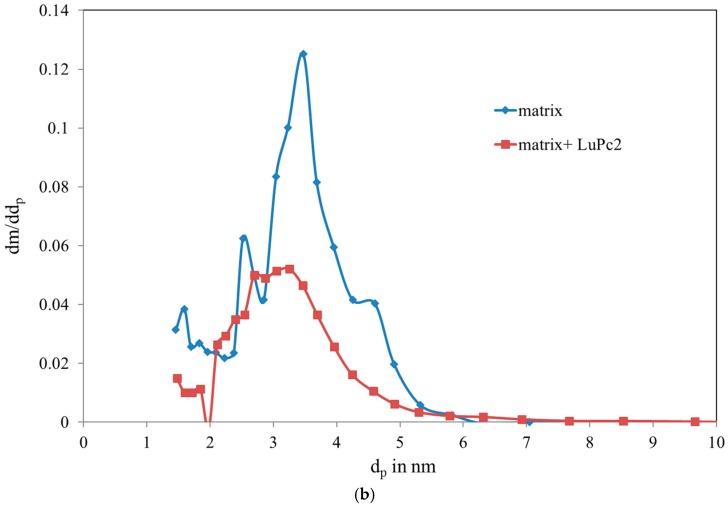
(**a**) Mass uptake during water adsorption and (**b**) pore size distribution of the matrix as a function of the pore diameter.

The porosity and pore size distribution of the films were measured using the adsorption isotherm of water. The evolution of the mass of the films during adsorption and desorption of water vapour was followed with a 6 MHz quartz microbalance. The results are presented in Figure 8a,b.

Figure 8a represents the relative mass uptake versus humidity. A saturation corresponding to the complete filling of the pores is visible at 29%. The corresponding total porosity ε is 37%.

The pore size calculated using Kelvin equation and BJH (Barret-Joyner-Halenda) method (Figure 8b) is in the range 2–5 nm, which is consistent with the TEM images. The specific surface area calculated by the BET (Brunauer-Emmet-Teller) theory is 310 m^2^/g. The calculation methods are explained in [42].

Figure 8b also shows the difference in pore size after impregnation of LuPc_2_. A decrease of the mean pore size is observed. According to the mass variation after impregnation (measured by the microbalance), it can be estimated that the amount of LuPc_2_ in the pores is 30% of the expected mass if the pores were completely filled. A plausible explanation of the incomplete filling is that the accumulation of the molecules on LuPc_2_ in the necks between the pores can block the other molecules trying to reach the centre of the pore.

### 3.2. Optical Characterization on Flat Substrates

**Figure 9:**
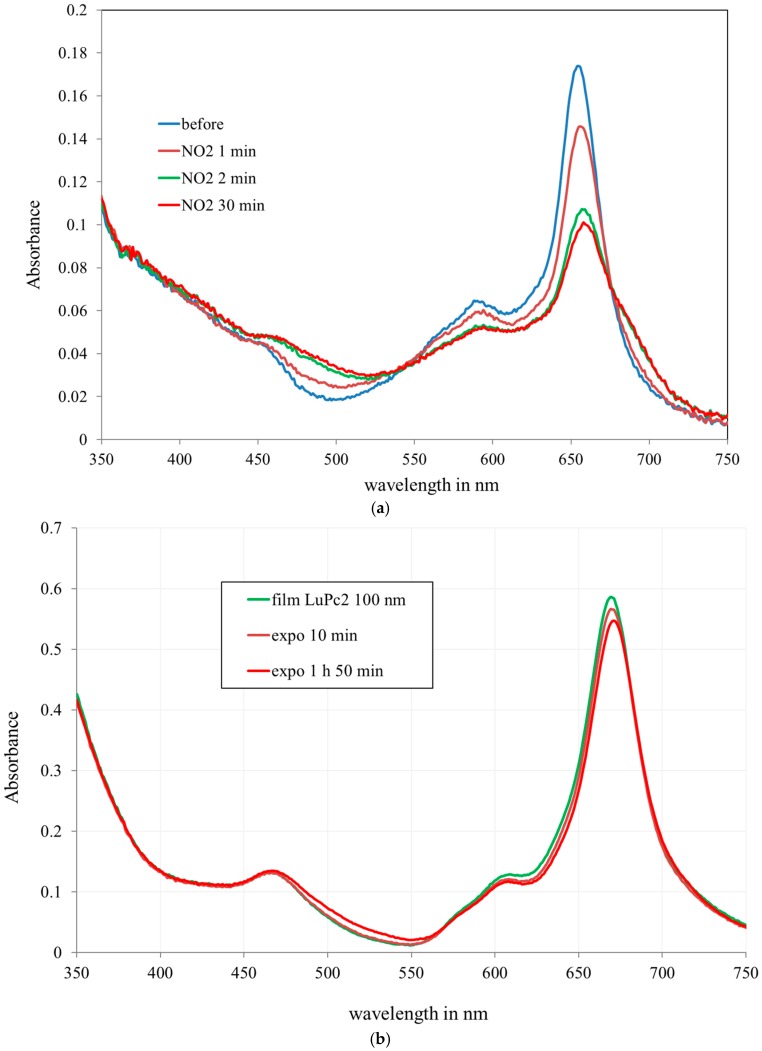
(**a**) Spectra of LuPc_2_ incorporated in the silica matrix on glass substrates after exposure to NO_2_ at 10 ppm in humid air (50% @ 20 °C). (**b**) Absorption spectra of a 100 nm LuPc_2_ film evaporated on glass substrates after exposure to NO_2_ at 10 ppm (after 10 min and 1 h 50 min).

The films deposited on flat glass substrates (microscope glasses) were exposed to 10 ppm NO_2_ in air (Figure 9a) and compared with films of pure LuPc_2_ deposited by thermal evaporation (Figure 9b). Figure 9a,b present the spectra of the mesoporous layer and the evaporated layer.

The absorbance* of the film was measured at 661 nm (corresponding to the maximum) (A = 0.18) and compared to the absorbance of a pure 100 nm film deposited by evaporation (A = 0.55) (Figure9b). As the film thickness is 320 nm, it was found that the filling according to these measurements is 29%, which is close to the evaluation by mass measurement.

* The absorbance is defined as A = −log(T) T is the ratio of transmitted light intensity/incident light intensity. A is dimensionless.

Although the filling is not complete, the films show a good sensitivity to NO_2_. The absorbance decreases by ca. 70% after only 2 min under 10 ppm NO_2_, for the LuPc_2_ incorporated in the silica matrix (Figure 9a), whereas it decreases only slightly after 10 min exposure at the same concentration for the 100 nm-thick LuPc_2_ film (Figure 9b).

**Figure 10:**
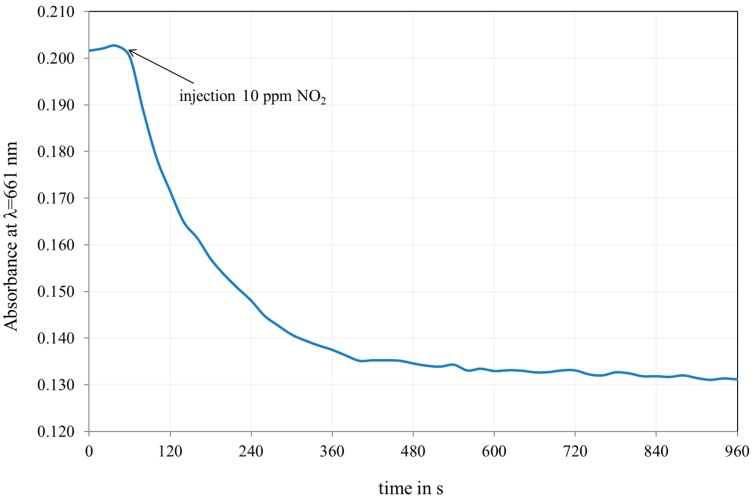
Evolution of the absorbance at 661 nm of a film exposed to 10 ppm NO_2_.

It can be clearly observed that the response time of the film in the silica matrix is drastically reduced. Figure 10 shows the evolution of the film when exposed to NO_2_. The absorbance decreases rapidly. After 6 min, the response is almost stable.

### 3.3. NO_2_ Sensing on Fibre

The sensitive layer was deposited in the same conditions as the reference glass substrates on the tip of a fibre. 

For the gas sensing tests, the reference baseline gas is synthetic air @ 50% RH @ 20 °C.

The sensor was put in the cell described in Section 2. The total gas flow rate is kept at 1000 mL/min. Before the injections, the sensor was left for at least 30 min with UV injection switched on in humid air (50% RH @ 20 °C) until the baseline is stable. 

**Figure 11:**
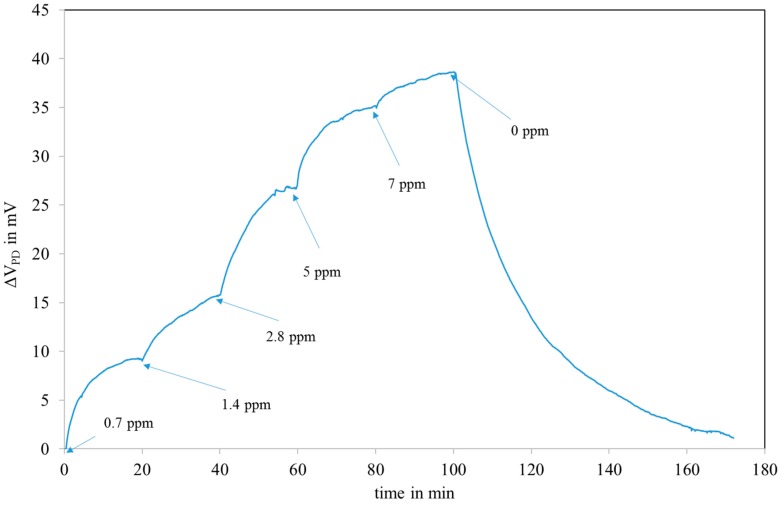
Response to NO_2_ injections in humid air (50% RH at 20°C) of a sensor covered with the LuPc_2_ impregnated mesoporous matrix.

Figure 11 shows the response of the fibre optic sensor to successive NO_2_ injections, from 0.7 to 7 ppm. The response Δ*V_PD_* is the output voltage of the photodiode in mV (zeroed on reference air). The UV injection is kept constant throughout the test (only switched off for the measurements with the red LED). The system is fully reversible after a recovery period comparable to the exposure period duration.

The test was carried out with 3 other sensors with similar responses (45 ± 10 mV for 7 ppm).

**Figure 12:**
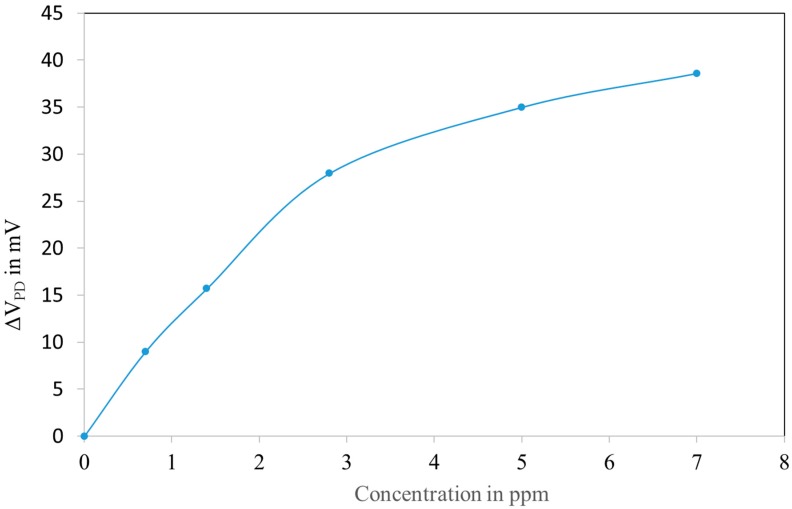
Photodiode voltage versus NO_2_ concentration in humid air (50% RH at 20 °C) of a sensor covered with the LuPc_2_ impregnated mesoporous matrix.

The interaction of the sensitive layer with NO_2_ reduces its absorption and as a consequence, the injections of NO_2_ lead to an increase of the reflected power (Figure 12). 

The reflected power saturates for concentrations above 10 ppm due to the saturation of the adsorption of NO_2_.

The response time t_90_ is about 6 min which is acceptable for air pollution monitoring. 

**Figure 13:**
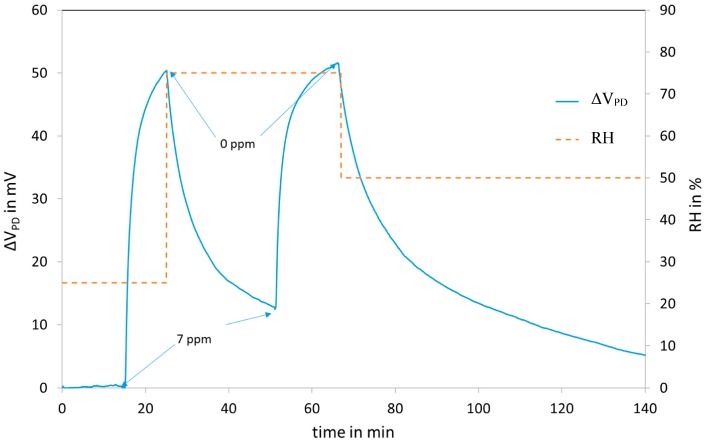
Effect of moisture on the reflectance of the sensor.

Figure 13 shows the effect of humidity on the response of the sensor. The humidity is varied from 25 to 75% @ 20 °C during the injection according to the cycle indicated by the dashed line. The humidity has a negligible effect on the response. 

Besides humidity, other possible interfering gases like H_2_, CO or NH_3_ were tested. No answer was observed for concentrations of 100 ppm. 

LuPc_2_ is well known to be sensitive to oxidizing gases like NO_2_ and Ozone in the low ppm range while the other gases like hydrocarbons, CO or VOC’s present negligible reactions except in high concentrations [44,45].

Ozone is very unstable and it is very easy to eliminate by a filter that selectively removes O_3_ while keeping NO_2_ unchanged [46,47]. 

### 3.4. Simple Model for the Sensor

The reflectance of the combination of the multimode fibre and the sensitive layer can be approximately described as the sum of the reflectance in the fibre-layer interface (*R_fl_*) and the reflectance in the layer-air interface (*R_la_*) (Figure 4):

(2) $$R\approx R_{fl}+R_{la}\cdot e^{-2\alpha d}$$

The light reflected at the layer-air interface experiences attenuation due to the absorption of the sensitive layer of length *d*, characterized by an absorption coefficient *α*, expressed in m^−1^. This coefficient is affected by NO_2_ exposure and the absorption decreases in presence of this gas (as seen in Figure 6).

The measurand related to the NO_2_ concentration is the photodetector voltage *V_PD_* as a result of the photodetected optical power reflected from the sensor. The photodetector voltage is given by (3):

(3) $$V_{PD}=P_{LED}\cdot {ℜ}_{PD}\cdot G_{PD}\cdot G_{T}\cdot R\approx K\cdot (R_{fl}+R_{la}\cdot e^{-2\alpha d})$$

where:

*P_LED_* is the output power of the red LED (emitting at 660 nm) in W;
${ℜ}_{PD}$ is the responsivity of the photodiode in A/W;
*G_PD_* is the transimpedance gain of the photodiode in V/A;
*G_T_* represents the optical losses and is lower than 1.

All these terms are constant and put together in constant *K*.

Equation (2) shows that *V_PD_* depends on the reflectance, since the other parameters are constant and *V_PD_* directly depends on the absorption of the layer. When *α* decreases due to interaction with NO_2_, *V_PD_* increases.

The refractive indexes of the fibre, the mesoporous layer and the absorption coefficient are known. The refractive index of humid air is taken as 1.

Fibre n_1_ = 1.45

Sensitive layer n_2_ = 1.33 (calculated by Lorentz-Lorenz theory and confirmed by measurements)

Absorption coefficient for the film *α* = 1.30 × 10^6^ m^−1^ (not in contact with NO_2_). This coefficient is easily calculated from the absorption spectra in Figure 8a.

The thickness of the layer is 320 nm.

Coefficients *R_fl_* and *R_la_* can be approximately calculated by

(4) $$R_{fl}={\left|\frac{n_{1}-n_{2}}{n_{1}+n_{2}}\right|}^{2}\approx 1.86\times 10^{-3}$$

(5) $$R_{la}={\left|\frac{1-n_{2}}{1+n_{2}}\right|}^{2}\approx 20\times 10^{-3}$$

So, Equation (3) becomes

(6) $$V_{PD}\approx K\cdot (R_{fl}+R_{la}\cdot e^{-2\alpha d})\approx K\cdot 10^{-3}\left(1.86+20e^{-2\alpha \left(C\right)d}\right)$$

The first term *R_fl_* is at first approximation independent of the NO_2_ concentration.

Absorption coefficient α(C) is a function of the NO_2_ concentration.

In our case, *α* = 1.30 × 10^6^ m^−1^ for NO_2_ = 0 and 0.68 × 10^6^ m^-1^ for NO_2_ = 10 ppm. 

If we take the reflectance in air without NO_2_ as the base line. The useful signal is the difference Δ*V_PD_* between air to measure and baseline.

(7) $$\Delta V_{PD}\approx K\cdot R_{la}\left(e^{-2\alpha \left(C\right)d}-e^{-2\alpha_{0}d}\right)$$

In our case, *α* = 1.30 × 10^6^ m^−1^ for NO_2_ = 0 (*α*_0_) and 0.68 × 10^6^ m^−1^ for NO_2_ = 10 ppm. 

*K* = 1100 mV.

Based on this simple model, we can evaluate the evolution of the response of the sensor varying the thickness of the sensitive layer. Indeed, if the layer is thin, the absorption is small and the absorption change will not be visible. On the contrary, if the layer is too thick, all the light will be absorbed and only the reflectance at the fibre-layer interface will be measured. So, the response is expected to present a maximum.

**Figure 14:**
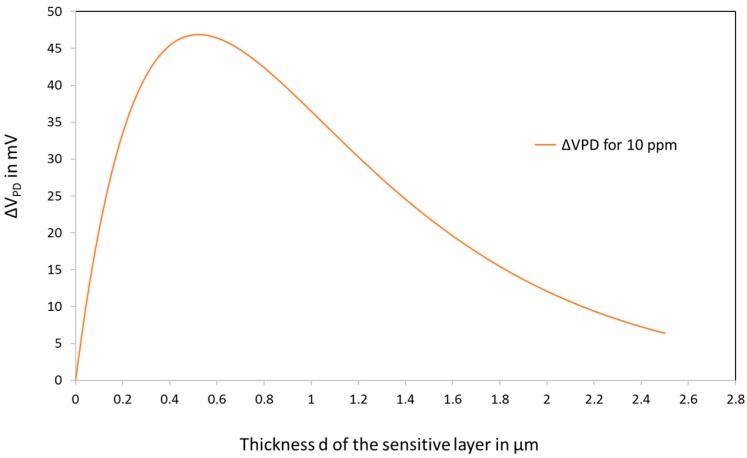
Effect of the thickness of the sensitive layer on the response of the sensor.

Figure 14 shows the theoretical evolution of the response of the sensor as a function of the thickness. A quite flat maximum is observed close to 500 nm. The 320 nm films that were prepared are in the good range. 

For practical applications, the fact that the response is not drastically influenced by the thickness fluctuations, at least in the range (0.2–1 µm), is an advantage. Indeed, during the manufacturing of the sensors, some tolerances on thickness, porosity, pore filling have to be taken into account but the most stringent is the control of the thickness. 

In practice, the sensors have to be calibrated to take into account the manufacturing tolerances and the tolerances in the optical circuit as well. The calibration simply consists in putting the sensor in a gas chamber and measure the response at 3 gas concentrations. Notice that for concentrations <3 ppm, the response is almost linear (slope typically 10 mV/ppm).

Assuming a simple Langmuir model for the evolution of the absorption coefficient, we can write:

(8) $$\alpha \left(C\right)=\alpha_{0}\cdot \left(1-\frac{k_{1}\cdot C}{k_{2}+C}\right)$$

**Figure 15:**
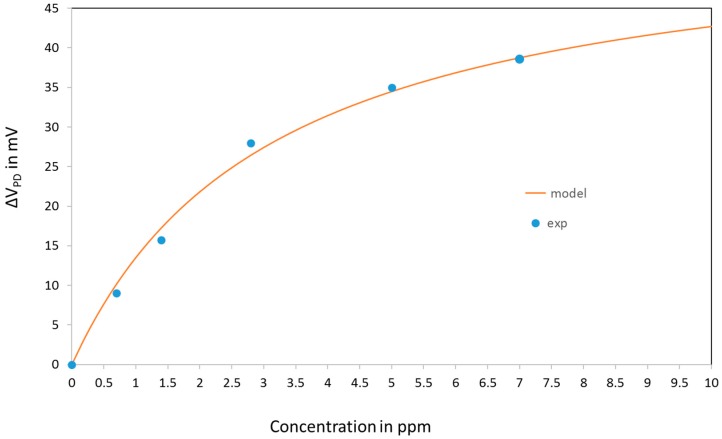
Simulation of the response of the sensor. Fitting of the model with the experimental data.

This simple model has been compared to the experimental data. The comparison is presented on Figure 15.

The coefficient *k*_1_ and *k*_2_ in Equation (8) were obtained by fitting with the experimental data.

## 4. Discussion

We presented here a reversible optical fibre sensor able to monitor NO_2_ in the concentration range (0–5 ppm) as required by the current legislations explained above. The detection limit is 50 ppb. The response time t_90_ is typically 10 min and the recovery time typically 20 min. This is compatible for pollution monitoring as the concentrations level change slowly in practice.

The main advantages of this kind of sensor are related to the use of optical fibres which are immune to electromagnetic noise and allow a remote sensing over long distances (useful for tunnels).

This sensor does not aim at replacing the commercial analysers based on chemiluminescence, which are very sensitive and accurate but also very expensive or cumbersome [48]. 

The performance of this sensor is comparable to the electrochemical cells that are sensitive in the same concentration range [49,50]. Sensors based on semiconductors show a lower detection limit and a better response time but are often limited by the lack in selectivity as they suffer of inferences by other exhaust gases [51].

## 5. Conclusions

This paper presents an optical fibre NO_2_ sensor based on LuPc_2_ encapsulated in a mesoporous silica matrix. The aimed detection range is (0–10 ppm). The sensor consists in a regular mesoporous silica matrix deposited by dip-coating on the tip of a fibre impregnated by LuPc_2_ in solution. The contact with NO_2_ drastically and reversibly decreases the optical absorption of the film at 660 nm. Due to a strong interaction with NO_2_, the LuPc_2_ thin films show a good sensitivity at low concentrations. It was confirmed that the porous matrix allows a better diffusion of the gases by blocking the growth of the LuPc_2_ crystals compared with the bulk material. It drastically reduces the response time t_90_ which is about 6 min. However, the recovery is rather slow. To reduce the latter, the films were irradiated by UV light at 365 nm in order to promote the photodissociation of the complex formed between NO_2_ and LuPc_2_ molecules. A multimode fibre was selected in order to allow the transmission of both UV and visible light through the fibre to illuminate the films. Doing so, the sensors show acceptable response and recovery times for pollution monitoring. The obtained sensor can be of practical interest for traffic pollution monitoring.
